# Intracellular Temperature Sensing: An Ultra-bright Luminescent Nanothermometer with Non-sensitivity to pH and Ionic Strength

**DOI:** 10.1038/srep14879

**Published:** 2015-10-08

**Authors:** Helin Liu, Yanyan Fan, Jianhai Wang, Zhongsen Song, Hao Shi, Rongcheng Han, Yinlin Sha, Yuqiang Jiang

**Affiliations:** 1 Single molecule & Nanobiology Laboratory, Department of Biophysics, School of Basic Medical Sciences and Biomed-X Center, Peking University, Beijing 100191, China; 2 State Key Laboratory of Molecular Developmental Biology, Institute of Genetics and Developmental Biology, Chinese Academy of Sciences, Beijing 100101, China

## Abstract

Luminescence thermometry usually suffer from cellular complexity of the biochemical environment (such as pH and ionic strength), and thus the accuracy and reliability of the determined intracellular temperature are directly affected. Herein, a photoluminescent nanothermometer composed of polymer encapsulated quantum dots (P-QD) has been developed. And the prepared nanothermometer exhibits some advantages: such as non-sensitivity to pH and ionic strength, as well as high detection sensitivity and ultrahigh reversibility. The intracellular temperature was accurately determined under physiological conditions with different pH and ionic strength, and direct measurement of thermogenesis in individual cells has been achieved.

Temperature sensing is important in many fields, including nano/microelectronics, biology and clinical diagnostics^1^. Different thermometers based on various temperature-dependent properties have been developed. Among of them, luminescence thermometry, using the temperature-dependent optical properties (e.g., emission intensity, emission peak position or decay lifetime) from luminescent materials to determine temperature, is particularly suitable for biological temperature sensing because of its non-invasive and inherently parallel characteristic. So far, different kinds of luminescence thermometry have been developed and some of them have been successfully applied for cellular temperature sensing^2^.

Quantum dots (QDs), combined with superior properties, such as excellent photostability and high brightness^3^,^4^, are excellent candidate materials for temperature sensing. Actually QDs-based thermometry has been widely developed. And the sensing scheme is generally based on the emission intensity or the decay lifetime^2^. For the emission intensity mode, dual-emission QDs are usually used to eliminate the effect of concentration, photobleaching and backgroud noise from biological specimens^2^. For example, dual-emission Mn^2+^-doped QDs probes have been developed and successfully applied for ratiometric celluar temperature sensing^5^,^6^.

A fundamental fact is that the pH and ionic strength within a live cell are highly varying^7^, and the emitting materials (such as QDs) usually change their optical properties (e.g., emission intensity, peak position and decay lifetime) depending on these environments. And thus, no matter what kind of sensing scheme involved, these biochemical factors (such as pH and ionic strength) are difficult to be delineated from the thermal effect and finally the accuracy and reliability of the determined intracellular temperature are directly affected^8^,^9^. Luminescence thermometers with high sensitivity to temperature and meanwhile non-sensitivity to physiological pH and ionic strength, are urgently needed for accurate and reliable sensing of intracellular temperature. Until now, synthesis of such a luminescence thermometer is technically challenging and only a few successful examples have been reported^10^,^11^.

Recently, we have developed a novel polymer-encapsulated QDs (P-QD), characterized by encapsulation of CdSeS/ZnS QDs in the matrix material of poly(methyl methacrylate-co-methacrylic acid) (PMMA-co-MAA)^12^. In contrast to previous reports of polymer-encapsulated QDs, the photoluminescence (PL) quantum yield (QY) of P-QD is highly enhanced as a result of the efficiently blocking nonradiative decay pathway from the surface trap state^12^. Interestingly, we found that P-QD exhibited strong resistance against physiological pH and ionic strength^12^.

Herein, a luminescence nanothermometer based on P-QD, has been developed and the prepared nanothermometer exhibits a linear response of PL intensity, with a high sensitivity. Notably, these nanothermometers show non-sensitivity to pH and ionic strength, as well as ultra-high reversibility. Intracellular temperature sensing with high accuracy and reliability, and direct measurement of thermogenesis in individual cells have been achieved with these P-QD as probes.

## Results and Discussion

**Figure 1:**
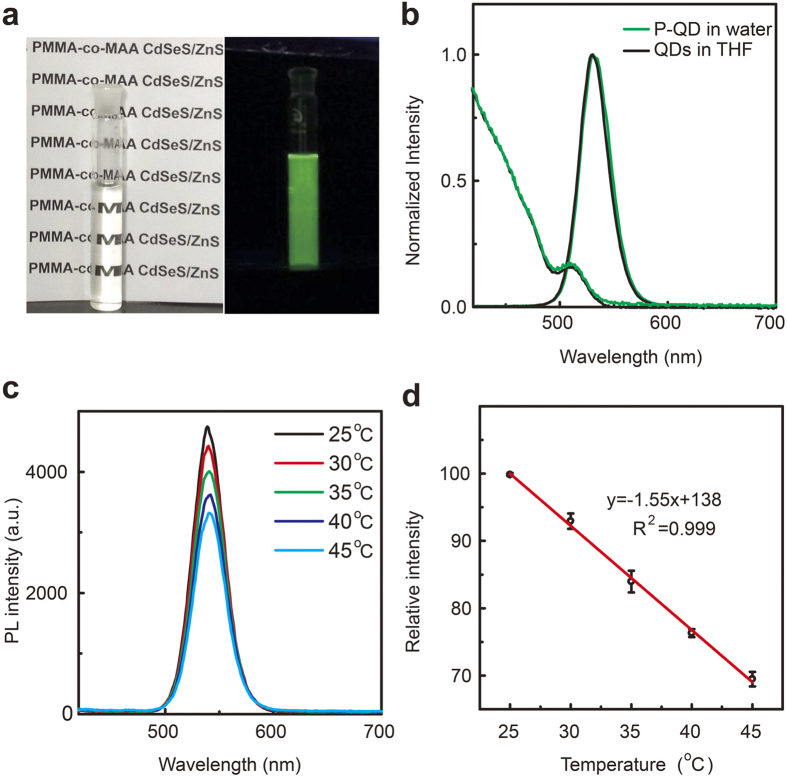
(**a**) Photograph of P-QD in water taken under natural light (left) and 365 nm light (right). (**b**) UV-vis absorption and photoluminescence spectra (*λ*_ex_ = 400 nm) of P-QD in water and initial QDs in THF. (**c**) Photoluminescence spectra of P-QD under various temperatures. (**d**) The PL intensity of P-QD as a function of temperature. Error bars represent standard deviations obtained from three parallel experiments.

The P-QD was synthesized via our previously developed co-precipitation-assembly method^13^. Figure 1a shows the prepared P-QD dispersed in aqueous solution that is highly transparent under natural light and emits green luminescence under ultraviolet irradiation (*λ*_ex_, 365 nm). As shown in Fig. 1b, both absorption and emission spectra are well matched, comparing QD with P-QD. These results indicate that QD structure and optical property are well preserved after the encapsulating process. Figure 1c shows that the PL intensity of P-QD decreases upon raising the temperature from 25 to 45 °C. And no apparent emission shift (<2 nm) observed, indicating that no photodegradation of QDs occurred during this experiment^14^,^15^. Further analysis reveals that the PL intensity decreases linearly (Fig. 1d), exhibiting a correlation coefficient (R^2^) of >0.999, with increasing temperature from 25 to 45 °C. The response sensitivity is determined to be −1.55% per °C, which is better than the values of current QDs-based nanothermometers with the similar detecting scheme^10^,^16^,^17^. The detection limit was determined to be 0.43 °C based on 3σ, where σ is the standard deviation of the PL intensity of P-QD at 25 °C. This high temperature resolution is comparable to or better than the resolution of current fluorescent thermometers^1^,^18^,^19^,^20^,^21^. Notably, the temperature resolution of our system is high enough for intracellular temperature mapping^22^.

**Figure 2:**
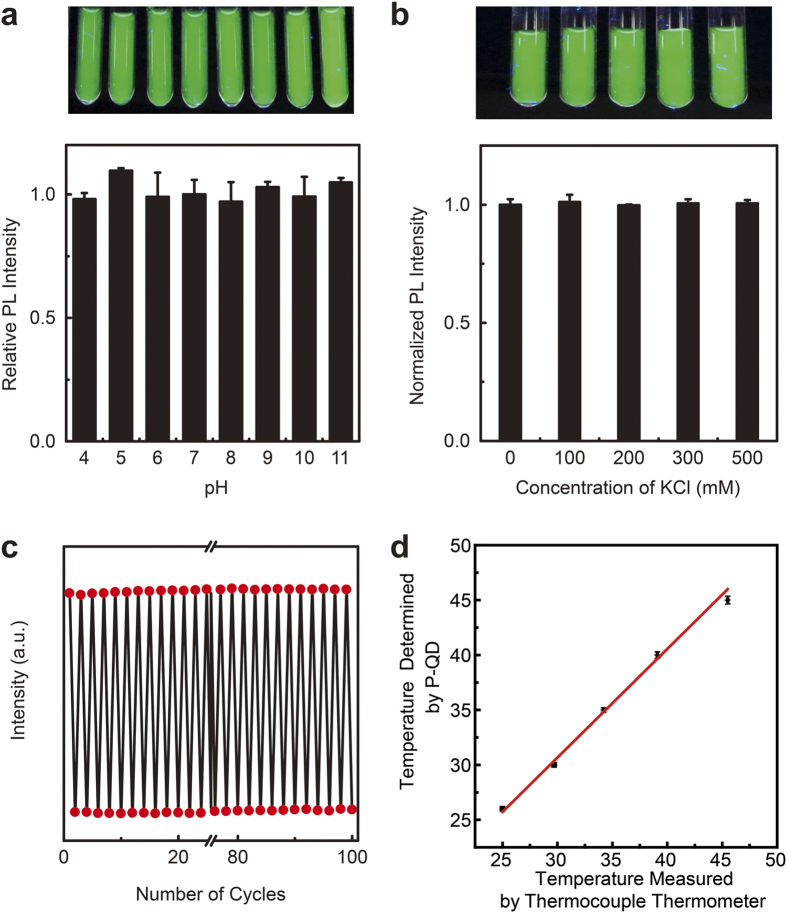
(**a**) Top: Photograph of P-QD in solutions with various pH, under 365 nm light. Bottom: The PL intensity as a function of pH. Error bars represent standard deviations obtained from three parallel experiments. (**b**) Top: Photograph of P-QD in solutions with different KCl, under 365 nm light. Bottom: The PL intensity as a function of the concentration of KCl. Error bars represent standard deviations obtained from three parallel experiments. (**c**) The PL intensity of P-QD under 25 °C (top panel) and 37 °C (bottom panel). The heating-cooling cycles were repeated 100 times. (**d**) The temperature determined by P-QD temperature probes and the values measured with a thermocouple thermometer. Error bars represent standard deviations obtained from three parallel experiments.

As mentioned above, luminescent thermometer should be insensitive to physiological pH and ionic strength. Figure 2 shows the PL intensities of P-QD at different pH levels and KCl concentrations and corresponding PL photographs taken under *λ*_ex_ 365 nm. Notably, P-QD in water exhibits strong PL emissions in a wide pH range of 4–11, and spectroscopic measurements indicate that PL intensity varies < 2.0% (Fig. 2a). We attribute this robust pH stability to the shielding effect of polymer encapsulation and the plentiful surface-covered carboxyl groups, which act as a protective shell around the nanoparticle. Figure 2b shows the effect of KCl on the optical properties of P-QD. Even when the concentration of KCl is up to 500 mM, much higher than the physiological ionic strength (~100 mM)^23^, the PL intensity still keeps unchanged and only 2% variation was observed.

Beside the non-sensitivity to pH and ionic strength, P-QD also exhibits a strong reversibility. As shown in Fig. 2c, even after 100 heating-cooling cycles between 25 °C and 37 °C, P-QD still remains luminescent and no apparent change of PL intensity was observed. The reversible PL response is higher than previously reported work (8 cycles^17^). Further study indicated that, in compared with the original QDs in THF, the photostability of P-QD was greatly enhanced (Figure S1), which can be ascribed to the shielding effect of polymer encapsulation. These results demonstrate the strong reversibility of P-QD and excellent photostability.

To further reveal the potential effect from intracellular components, we explore the temperature of a cell lysis solution, which contains nearly all cellular components, with P-QD as probe. As shown in Fig. 2d, nearly perfect correlation is observed for the measured values using the P-QD sensor and the real values (measured with a thermocouple thermometer). Based on these above results, such as the non-sensitivity to pH and ionic strength, combined with excellent reversibility and rather high response sensitivity, we conclude that P-QD is an ideal probe for accurately monitoring intracellular temperature.

**Figure 3:**
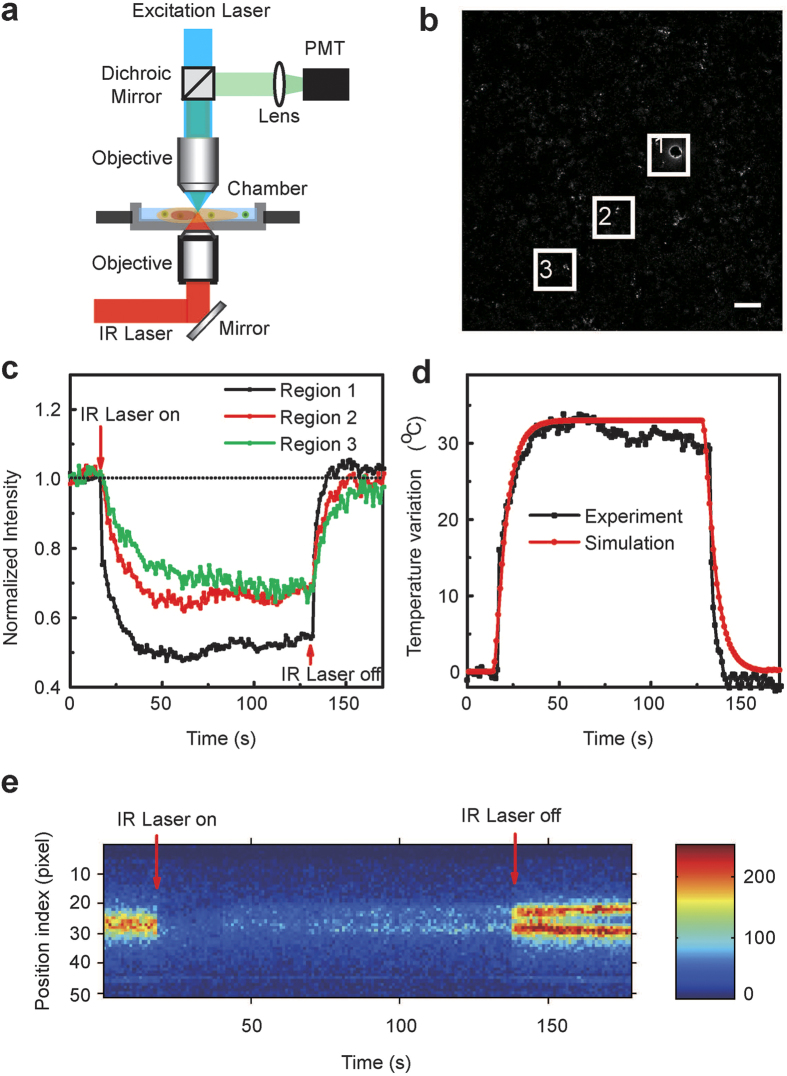
The temperature experiment controlled by infrared laser. (**a**) Schematic of the experimental setup, illustrating the essential optical pathways in a Zeiss LSM 780 confocal microscope. (**b**) Representative snapshot of P-QD treated HepG2 cells. Scale bar:100 μm. (**c**) The PL intensity of P-QD as a function of time for the region 1–3 shown in Fig. 3b. (**d**) Local temperature variations (Δ*T*) and simulation results as a function of time for the region 1 shown in Fig. 3b. (**e**) Location-dependent temperature changes within a live cell as a function of time for the region 1 shown in Fig. 3b. The time points at which IR laser was on/off are marked by an arrow. The uncertainties of position localization as estimated from the Gaussian fitting are represented as shades of the temperature colour; the more intense and the narrower the vertical distribution, the more accurately the location is.

For a more practical application, live cells response to the ambient temperature variation was investigated. As shown in Fig. 3a,c, when the ambient temperature was controlled by infrared laser, the PL intensity of P-QD varied with the temperature variation. And this response is sensitive and contemporaneous (Fig. 3c). When the IR laser was on, the PL intensity of P-QD decreased immediately. After about 30 s, the balance of temperature reached between the ambient and inside of cells. And then the IR laser was turned off, the PL intensity of P-QD could return to the initial intensity. The change rule of curve conforms to the law of thermodynamic (Fig. 3d). The whole process can be simulated by using the heat conduction equation with the parameters in the experiment^24^. The curve get from experiment is identical with the simulative curve. The results are in accordance with other studies^24^,^25^. The position-dependent temperature changes for a live cell as a function of time are shown in Fig. 3e. It is apparent that the temperature inside the cell increased when the IR laser on and decreased when the IR laser off. And different regions of the cell exhibit different temperature response. These results are consistent with previous studies^20^.

**Figure 4:**
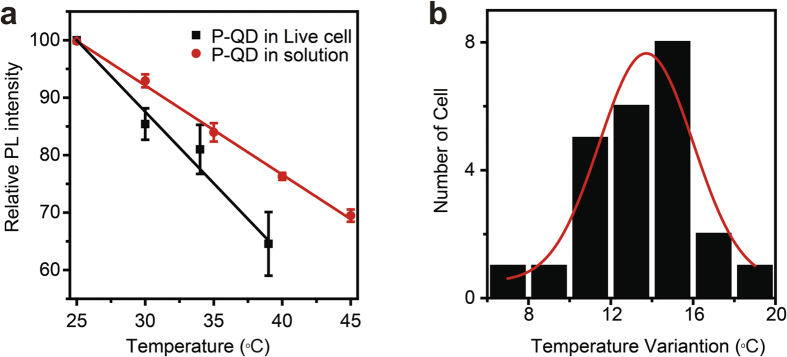
(**a**) The PL intensity of P-QD as a function of temperature in solution and live cells. Error bars represent standard deviations obtained from three parallel experiments. (**b**) Statistics of temperature variations for the 24 P-QD treated cells, showing a bigger variation value of 13.7 ± 0.44 °C than that of environment (9 °C). The red line is the Gaussian fitting curve.

When the ambient temperature was controlled by a metallic incubator, the PL intensity of P-QD within living HepG2 cells was recorded on a Zeiss LSM 780 confocal microscope. And the three-dimensional image indicates that P-QD was internalized into cells via non-specific pathways (Figure S2). As shown in Fig. 4a, when the ambient temperature is increased from 25 °C to 37 °C, for P-QD within living HepG2 cells, the slope of PL intensity-temperature curve is −2.49% per °C, much higher than that of P-QD in solution (−1.55% per °C). These above results suggest that live cells are more sensitive to temperature variation. Although the mechanism is not fully understood, we infer that the rate of cellular metabolism contributes to our results^26^. It is known that temperature changes affect biosynthesis and degradation, as well as the rate of enzymatic reaction^27^. Cells respond to a temperature increase by increasing metabolism, membrane fluidity, transcriptional activity, protein synthesis and vesicular transport rates. And further study indicates that when the ambient temperature increased from 25 °C to 34 °C (Δ*T*_ambient_ = 9 °C), the average intracellular temperature of 24 cells examined increased with Δ*T*_intracelluar _= 13.7 ± 0.44 °C (Fig. 4b). This direct observation of cellular thermogenesis is consistent with previous reports. Direct measurement of thermogenesis in individual cells is challenging because heat from thermogenerating organelles diffuses rapidly throughout the cell and beyond^28^. Recently, Yang *et* *al*. reported that non-homogeneous local temperature progression in NIH/3T3 cells following Ca^2+^ stress and cold shock^20^. Okabe *et* *al*. reported that the local temperature of the nucleus and centrosome of a COS 7 cell showed a significantly higher temperature than the cytoplasm^22^. More recently, direct mitochondrial thermogenesis was also observed^26^,^29^. Although these previous advance, living cells respond to a temperature variation has not been fully understood, especially at the single cellar level. As for our system, the increase of the rate of cellular metabolism can contribute to our results^26^, just as discussed above.

## Conclusions

In summary, a luminescence nanothermometer based on P-QD, has been developed. And the prepared nanothermometer exhibits non-sensitivity to pH and ionic strength, as well as high detection sensitivity and ultrahigh reversibility. These advantages are highly favorable and desirable for the accuracy and reliability of intracellular temperature determination. Based on the prepared nanothermometer, the intracellular temperature was accurately determined and direct measurement of thermogenesis in individual cells has been achieved. The encapsulation strategy demonstrated in the present work can be easily extended to the fabrication of other QDs-polymer hybrid luminescent nanoparticles, such as dual-emission system, for ratiometric intracellular temperature sensing.

## Methods

### Synthesis of CdSeS/ZnS QDs

The CdSeS/ZnS QDs used were prepared according to the literature^30^,^31^ with minor modifications. Briefly, CdO (0.05 g), oleic acid (OA, 0.46 g), and tri-n-octylamine (TOA, 15 mL) were mixed in a three-neck round-bottom flask and heated to 300 °C to get a clear solution under argon. A stock solution of Se (0.0021 g) and S (0.0124 g) in trioctylphosphine (1.0 mL) was swiftly injected into the hot solution of CdO/OA/TOA and allowed the reaction to proceed at 280 °C for 1 min. The system was cooled down to 150 °C and then heated to 240 °C, while the mixture of ZnO (0.5 mmol) in OA stock solution (1.0 mL) and S (0.5 mmol) TOP solution (1.0 mL) was titrated into the flask and allowed the reaction proceed at 240 °C for 1 minute. The CdSeS/ZnS QDs were obtained via precipitation with ethanol, washed, and dried in a vacuum oven for further studies.

### Preparation of P-QD

Typically, a THF solution (1.0 mL) containing CdSeS/ZnS QDs (1.0 mg) and PMMA-co-MAA (1.0 mg) was dropwise added into water (10.0 mL) under stirring (~100 rpm) at room temperature. The mixture was stirred for about 10 min, yielding a transparent colloidal solution. THF in the colloidal solution was removed at 45 °C by vacuum-rotary evaporation. Then P-QD was obtained via centrifugation at 50,000 g and re-dispersed under ultrasonic conditions in 10.0 mL of pure water.

### Characterization of Optical Properties

UV-vis absorption spectra were performed on a UV-vis spectrometer (TU-1901, Purkinje General Co., Ltd, Beijing, China). PL spectra of P-QDs in water and THF were recorded by a spectrophotometer (F-4500, Hitachi Co., Ltd, Japan). P-QDs was excited at 400 nm, and the PL spectrum was collected from 400 to 700 nm.

PL spectra of P-QDs were recorded from 25 to 40 °C at intervals of 5 °C with an accuracy of ±0.1 °C. The temperature was controlled by a metallic incubator (H_2_O^3^-PROIII, Coyote Bioscience Co., Ltd, China) and monitored by a digital thermocouple (TM-902C, Apuhua Co., Ltd, Shenzhen, China) with an accuracy of ±0.1 °C.

The pH of the solution (pH 4.0–11.0) was adjusted using HCl or KOH as monitored by a pH meter (PB-10, Sartorius Co., Ltd, China). Ionic strength concentration was regulated by KCL (0–500 mM).

Evaluate the thermal stability of P-QD in water by the PL intensity of P-QD under 25 °C and 37 °C. The heating-cooling cycles were repeated 100 times.

### Preparation of cell extract

The cell pellets (HepG2, ~5 × 10^6^) were collected and resuspended in RIPA Buffer (containing 25 mM Tris•HCl, pH 7.6, 150 mM NaCl, 1% NP-40, 1% Triton X-100, 1% sodium deoxycholate, 0.1% SDS). After gently shaking for 15 minutes on ice, the mixture was centrifuged at ~14,000 × g for 15 minutes. The supernatant was collected for further analysis.

### Cell Culture and cell imaging

HepG2 cells were propagated in DMEM supplemented with 10% fetal bovine serum (FBS) and 80 U mL^−1^ gentamycin sulfate. Then the cultured cells were trypsinized and re-suspended in this DMEM Medium at a concentration of about 7.5 × 10^5^ mL^−1^. The cell suspension (100 μL) was transferred to a confocal dish (35 mm). After incubation for 24 h at 37 °C in 5% CO_2_, the cells were carefully rinsed with PBS solution (pH 7.4). Then a colloidal solution of P-QD (50 μL, 0.1 mg mL^−1^) was added. After 2 h incubation at 37 °C in 5% CO_2_, the dish was rinsed three times with PBS solution (pH 7.4) and then 1 mL of fresh serum-free medium was added. The plates were incubated for another 10 minutes at 37 °C and then directly imaged on a Zeiss LSM 780 confocal microscope system equipment with Spectra-physics MaiTai Ti:sapphire laser (100 fs, 80 MHz repetition rate) and a water objective lens (40×).

The temperature was controlled by fs laser or a metallic incubator. (1) For the group of fs laser, the temperature was controlled by Spectra-physics MaiTai Ti:sapphire laser (100 fs, 80 MHz repetition rate). (2) For the group of metallic incubator, the temperature was controlled by a metallic incubator (H_2_O^3^-PROIII, Coyote Bioscience Co., Ltd, China) and monitored by a digital thermocouple (TM-902C, Apuhua Co., Ltd, Shenzhen, China) with an accuracy of ±0.1 °C. Record the change of fluorescence intensity from 25 to 40 °C at intervals of 5 °C with an accuracy of ±0.1 °C.

## Additional Information

**How to cite this article**: Liu, H. *et al.* Intracellular Temperature Sensing: An Ultra-bright Luminescent Nanothermometer with Non-sensitivity to pH and Ionic Strength. *Sci. Rep.* **5**, 14879; doi: 10.1038/srep14879 (2015).

## Supplementary Material

Supplementary Information
