# Supplementary material for: Intracellular Temperature Sensing: An Ultra-bright Luminescent Nanothermometer with Non-sensitivity to pH and Ionic Strength
Source: Sci Rep. 2015 Oct 8;5:14879. doi: 10.1038/srep14879 (PMC4597201; doi:10.1038/srep14879)
Supplement: Supplementary Information [file srep14879-s1.doc]

Supporting Information

**Intracellular Temperature Sensing: An Ultra-bright Luminescent Nanothermometer with Non-sensitivity to pH and Ionic Strength**

*Helin Liu, Yanyan Fan, Jianhai Wang, Zhongsen Song, Hao Shi, Rongcheng Han*,*

*Yinlin Sha* and Yuqiang Jiang**^†^

H.L. Liu, J.H. Wang, and Prof. Y.L. Sha

Single molecule & Nanobiology Laboratory, Department of Biophysics, School of Basic Medical Sciences and Biomed-X Center, Peking University, Beijing 100191, China. E-mail: shyl@hsc.pku.edu.cn (Y.L. SHA).

Y.Y. Fan, Z.S. Song, H. Shi, Prof. R.C. Han, Prof. Y.Q. Jiang

State Key Laboratory of Molecular Developmental Biology, Institute of Genetics and Developmental Biology, Chinese Academy of Sciences, Beijing 100101, China. E-mail: yqjiang@genetics.ac.cn (Y.Q. JIANG); hanrch@genetics.ac.cn (R.C. HAN)

State Key Laboratory of Molecular Developmental Biology, Institute of Genetics and Developmental Biology, Chinese Academy of Sciences, Beijing 100101, China.

**Table S1.** Comparison of the sensitivity and temperature resolution of the present work and previously reported works.

| **Fluorescent thermometers** | **Sensitivity** | **Reference** |
| --- | --- | --- |
| Mn-doped core-shell nanocrystals | -0.5% per ^o^C | *Nanoscale* ***2013,*** *5 (11), 4944* |
| QDs | -1.3% per ^o^C | *Appl Phys Lett* ***2003,*** *83 (17), 3555* |
| CdTe QDs | -1.47% per ^o^C | *Chem Commun* ***2013,*** *49 (10), 969* |
| P-QD (CdSeS/ZnS) | -1.55% per ^o^C | The present work |

| **Fluorescent thermometers** | **Temperature resolution** | **Reference** |
| --- | --- | --- |
| Fluorescent nanogel thermometer | 0.29-0.5 ^o^C | *J. Am. Chem. Soc.,***2009,** *131*: 2766 |
| l-DNA molecular beacon | 0.7 ^o^C | *J. Am. Chem. Soc.*, **2012,** *134*: 18908 |
| QDs | 0.49-0.97 ^o^C | *ACS Nano*, **2011,** 5: 5067 |
| P-QD (CdSeS/ZnS) | 0.43 ^o^C | The present work |


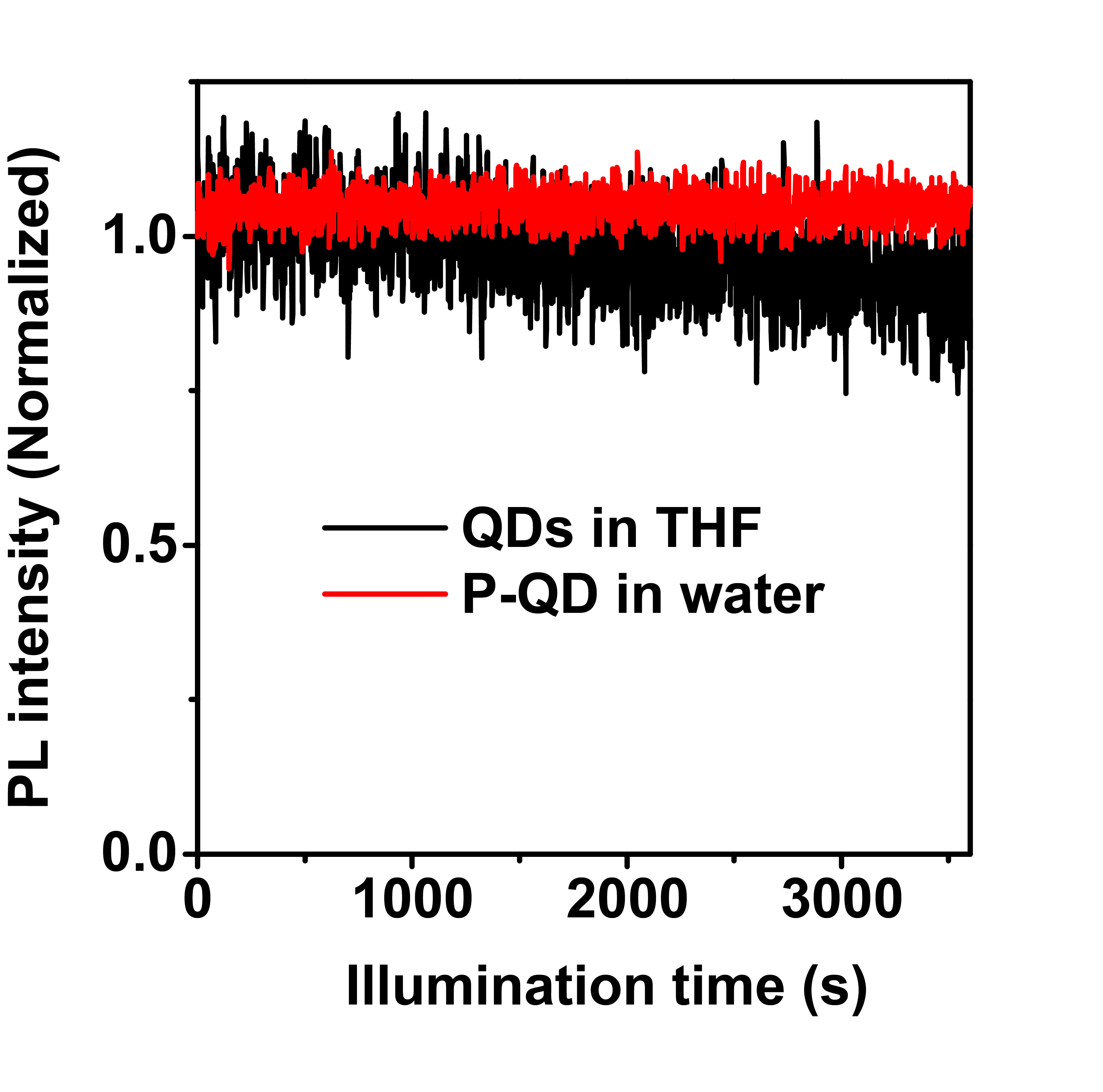


**Figure S1.** Time courses of PL intensity changes of P-QD in water and QDs in THF under the continuous illumination. The experiments were performed on an F-4500 fluorescence spectrophotometer (Hitachi，Japan) with Time Scan Mode (*λ*_ex_ = 400 nm, *λ*_em_ = 530 nm). The concentration of QDs and P-QD was tuned to be same with an absorption of 0.03. The split widths of excitation and emission were set at 2.5 and 2.5 nm, respectively. No extra protection against O_2_ was conducted.


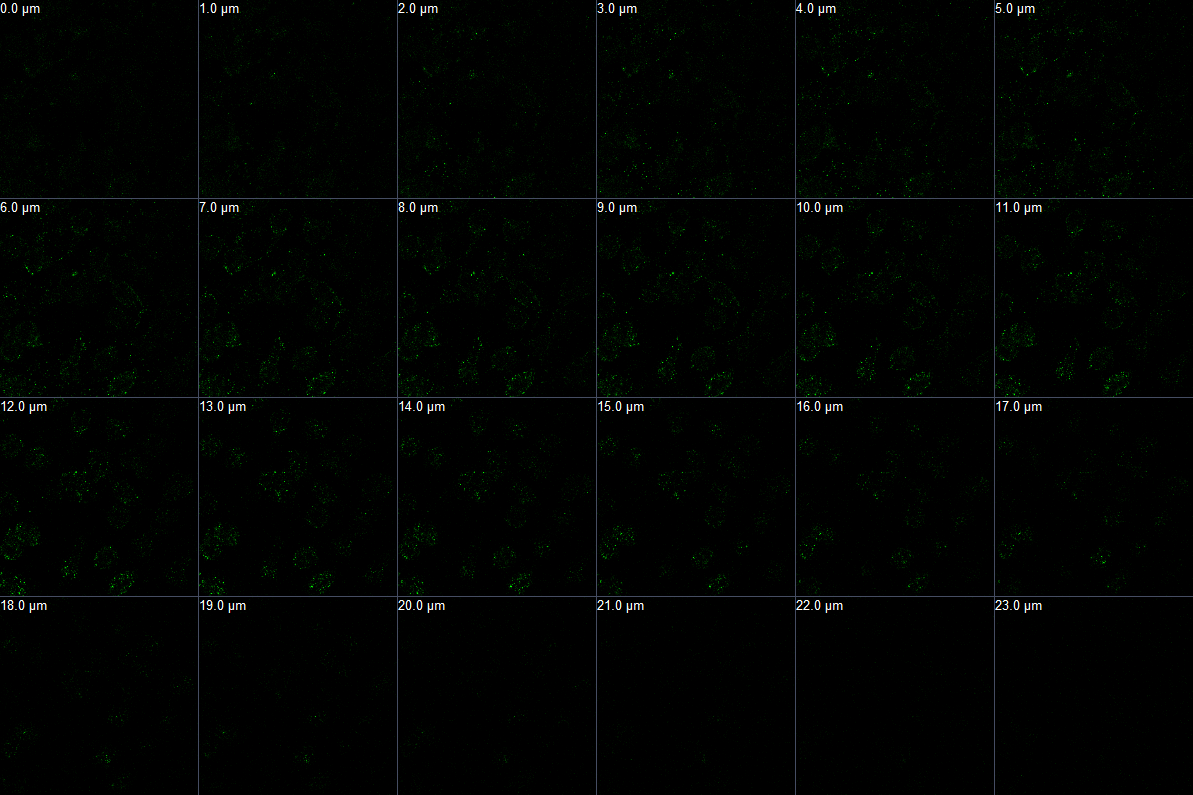


**Figure S2.** 3D imaging of P-QD-treated HepG2 cells. The three-dimensional image indicates that P-QD was internalized into cells via non-specific pathways.
